# Should Severity Assessments in Healthcare Priority Setting be Risk- and Time-Sensitive?

**DOI:** 10.1007/s10728-023-00460-0

**Published:** 2023-08-01

**Authors:** Lars Sandman, Jan Liliemark

**Affiliations:** Department of Health, Medicine and Caring Sciences, National Centre for Priorities in Health, Linköping University, Linköping, SE-581 83 Sweden

**Keywords:** Priority setting, Healthcare, Severity, Risk-sensitivity, Time-sensitivity, Discount

## Abstract

Background: Severity plays an essential role in healthcare priority setting. Still, severity is an under-theorised concept. One controversy concerns whether severity should be risk- and/or time-sensitive. The aim of this article is to provide a normative analysis of this question. Methods: A reflective equilibrium approach is used, where judgements and arguments concerning severity in preventive situations are related to overall normative judgements and background theories in priority-setting, aiming for consistency. Analysis, discussion, and conclusions: There is an argument for taking the risk of developing a condition into account, and we do this when we consider the risk of dying in the severity assessment. If severity is discounted according to risk, this will ‘dilute’ severity, depending on how well we are able to delineate the population, which is dependent on the current level of knowledge. This will potentially have a more far-reaching effect when considering primary prevention, potentially the de-prioritisation of effective preventive treatments in relation to acute, less-effective treatments. The risk arguments are dependent on which population is being assessed. If we focus on the whole population at risk, with T_0_ as the relevant population, this supports the risk argument. If we instead focus on the population of as-yet (at T_0_) unidentified individuals who will develop the condition at T_1_, risk will become irrelevant, and severity will not be risk sensitive. The strongest argument for time-sensitive severity (or for discounting future severity) is the future development of technology. On a short timescale, this will differ between different diagnoses, supporting individualised discounting. On a large timescale, a more general discounting might be acceptable. However, we need to also consider the systemic effects of allowing severity to be risk- and time-sensitive.

## Background

In healthcare priority setting, patients’ healthcare needs play an important role in guiding priority decisions in attempts to balance effectiveness against equity [1]. Thus, when we prioritise conditions for patients with great healthcare needs above patients with a lower degree of healthcare needs, we accept a higher threshold for cost-effectiveness and a larger opportunity cost. This paper is rooted in the understanding that severity is a central aspect of healthcare needs in many jurisdictions, as follows: [2].

P has a healthcare need for intervention Y if P benefits by moving from Z_current level_ toward Z_reference level_ through Y.

Severity is then defined as the gap between Z_current level_ and Z_reference level_, where Z signifies the objective of healthcare—i.e., health—as it is broadly understood. Hence, with a greater gap, and greater severity, there is greater healthcare need; ceteris paribus. This is how healthcare jurisdictions like the Netherlands, Sweden, and Norway—and their legalised frameworks for priority setting—seem to understand the concepts. This will also be the understanding on which we base the following analysis. For sure, severity might be somewhat differently understood in other frameworks for priority setting, and may not be as clearly related to healthcare need [3–5]. In such alternative understandings, the this article’s analysis might not apply. This might also, partly, explain the lack of consistency regarding how severity is interpreted and applied in different frameworks, and several issues are handled differently in different contexts [6, 7].

One issue of controversy, shown for example in how Sweden and Norway apply the concept, is how to assess severity in a situation of disease prevention or health promotion in healthcare settings; i.e., when the intervention concerns an individual’s future health, but where there are no current manifest illness or symptoms. Rather, the individual or a group of individuals are at risk of developing an illness later in life. Now, most would still think that the individuals in this situation are in need of preventive or promotional interventions—but how should these be factored into the above definition of severity? If we accept that there is a healthcare need for preventive or promotional interventions, Z_current level_ might not only signify manifest illness or symptoms, but also the need to incorporate the risk of future illness and/or the timing of future illness into the equation some way.

In healthcare practice, we can see two basic ways of addressing this issue. In Sweden, the official practice when it comes to assessing severity in preventive situations has been to assess the severity of the condition(s) we want to prevent, and then ‘downgrade’ this in relation to the risk of developing the condition in a given population [8]. Hence, if the severity of the condition to prevent is very high (on the four-grade scale used in Sweden: low, moderate, high, and very high), and the population has a 10% risk of developing this condition, the severity is set at a lower level (e.g., moderate). This has mainly been explained by reference to risk, but we also find references to time (in terms of discounting of future events) [8, 9]. Using the above terminology, we might say that Z_current level_ is risk- and/or time-sensitive, therefore Z_current level_ in preventive situations is defined as Z_potential level_ x (risk of developing the potential illness resulting in such a level, discounting for time). Later on, we will explain why it is important to distinguish between being risk- and time sensitivity.^1^

In Norway, a recent parliamentary committee resulted in a change to how severity is assessed [5]. In relation to prevention and promotion, it was decided that the assessment of severity should only consider those patients in an at-risk population who will develop the condition (and not the entire population) [5]. In essence, this model implies that assessing the severity of a specific condition in a preventive situation does not take population risk into account or discount for time. The main reason for this, as stated in the parliamentary decision, is to upgrade the priority of preventive interventions or to avoid giving preventive interventions lower priority [5]. Expressed in the terminology above, Z_current level_ in preventive situations in Norway is Z_future level of patients who will develop the condition_.^2^ Thus, this implies different understandings of the concept of healthcare need, and will impact on how preventive interventions are prioritised against other interventions in these jurisdictions. Despite this, there is no in-depth analysis of the normative arguments for or against these two different approaches.

The aim of this paper is to normatively analyse the arguments for and against different approaches to how risk and/or time might be taken into account when severity is assessed.

## Methods, Outline of the Paper, and Some Preliminary Remarks

In this paper, we will use a reflective equilibrium approach, where considered judgements and arguments concerning severity in preventive situations will be related to overall normative judgements and background theories in priority setting, aiming at consistency [10]. The paper will have the following outline: first we outline the arguments for and against risk-sensitive severity, then we examine the arguments for and against time-sensitive severity, and finally we examine potential systemic effects. The paper then ends with our conclusions and suggestions for addressing this issue going forward.

Before turning to the normative analysis, we need to say something about severity. As indicated in the introduction, severity (reflecting the healthcare needs of a population), has been described as an essentially contested concept, where there are a large number of unresolved issues and disagreements [6, 7]. For the analysis in this article, it is enough to accept the following two assumptions concerning severity:

- Taking severity into consideration implies assessing whether patients (or potential patients) in a specific situation are worse off than reference-level individuals in terms of maintaining good health.
- If a patient or patient group is in a more severe situation or has a more severe condition, this implies a greater claim on healthcare resources—since they have a greater healthcare need—than would be the case if a patient group was in a less severe situation or had a less severe condition.

One of the unresolved issues of severity is which aspects—exactly—to include in a severity assessment. In both the Norwegian and Swedish jurisdictions (as in many other jurisdictions), severity refers to a combination of impact on health-related quality of life (HRQoL) and mortality. However, exactly what to include in HRQoL, how to measure this, how to include the condition’s duration, how to balance this against mortality, and where the reference level is situated against the condition being assessed all vary [6].

Since exactly how to assess severity is not relevant to our principled argument, let us assume that severity varies from 0 to 100, where 100 is the worst possible severity (whatever that implies in terms of impact on mortality and HRQoL ), and 0 implies no severity (i.e., a level of health in line with the reference level).

## Analysis and Discussion

### Arguments for and Against Risk-Sensitive Severity

**Fig. 1:**
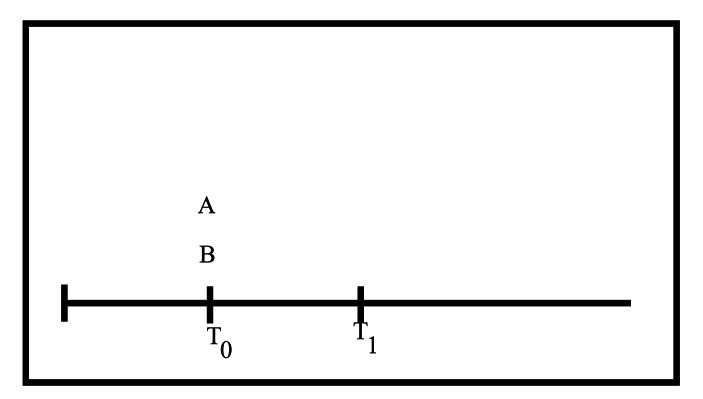
Illustration of two patients, one with a developed condition (A) at T_0_, and one with a risk at a time T_0_ of developing the same condition at a later time T_1_ (B)—where T_0_ and T_1_ have a significant amount of time between them (i.e., years rather than minutes)

Starting with risk-sensitivity, we will assume that if severity is risk-sensitive, this implies that the severity of the condition we are considering should be ‘downgraded’ to some extent in relation to the risk a population has of developing this condition.^3^

Consider Fig. 1 and imagine the following case:

#### Case 1

Person A is at T_0_ suffering from condition X with severity 100. Person B is at T_0_ and has a 10% risk of developing condition X at T_1_ (significantly distant in time from T_0_). Which of these persons are worst off *at T*_*0*_?

Most of us probably have the intuition that person A is worse off than person B at T_0_; i.e., having actually developed a negative condition is worse than only having a risk (< 100%) of developing the same condition. If we accept this intuition it implies that A has a greater claim on scarce resources than B at T_0_ and a higher priority if resources are limited—we might only be able to help A but not B. One way to operationalise this would be to say that B has a severity of 10 = 0.1*100 at T_0_ (we will return to potential problems with such an operationalisation later). Note that we, at this point, do not take time into account, and therefore the fact that condition X might affect B at T_1_ instead of at T_0_ is not taken into account (see below). This intuition also implies that the following is true:

Person A (having developed condition X) has the same severity as Person C (with a 100% risk of developing condition X at T_1_).

Persons A and C both have worse conditions than Person D (with a 50% risk of developing condition X at T_1_) which in turn, has a worse condition than Person B (with a 10% risk to developing condition X at T_1_).

Normally, when we prioritise resources in healthcare, we do not look at individual patients but at populations. So, what happens if we scale up the above reasoning to populations, as illustrated in Case 2?

#### Case 2

Population A (n = 10) at T_0_ is suffering from condition X at severity 100. Population B (n = 100) at T_0_ has a low risk of developing condition X at T_1_. Which of these populations are worse off?

We might set the risk for population B in this example so that the total disease burden or severity in both populations will be the same over time. However, normally, when using severity to guide priority setting, we do not take into account the total severity of a population, but rather consider the mean or median severity of the population. If not, a large population with a mild condition would have a higher total severity than a small group with a very severe condition.

Accepting this, if all members of population B have an equally low risk of developing X at T_1_, the conclusion from Case 1 follows logically. In a more realistic scenario, the risk of developing X is distributed differently in population B—ranging from some individuals having a small risk of developing X and some having a higher (and perhaps very high) risk of developing X—perhaps patterned along a standard normal distribution. Would we still claim that the severity of population B is lower than that of population A? As a population they do have a lower mean or median risk of developing X, but some individuals in population B might have a 100% risk of developing X. The problem is simply that we have not yet identified the factors determining this—e.g., the genetic combination that, when combined with environmental or situational factors, will cause X. At the same time, there might be genuine indeterminacy even given perfect knowledge of exactly which patient will develop X. We might imagine the following situation:

#### Case 3

Population B (n = 100) has a low mean risk of developing condition X. New diagnostic tools now enable us to narrow down the population to B_narrow_ with 20 individuals having a high mean risk of developing condition X, and where the other 80 individuals of population B have close to 0 risk of developing the condition.

The reduced population of B_narrow_ will therefore have a greater claim for resources than the original population B (since they will have a higher mean severity if we allow severity to be risk-sensitive). With better knowledge, we might provide a more distinct assessment of the risk (either higher or lower); e.g., recent developments in epigenetics might change our assessment from risks based on epidemiological studies to more individualised risks [11]. We might find it troubling that a lack of knowledge affects the severity assessment of a population. However, when assessing the severity of a population with a manifest condition, we also assess the mean severity of the group. In population A, all individuals suffered from the worst severity ever, therefore there is no variation, but in any other (real) population there will be variation. With increased knowledge, we might be better able to differentiate between sub-populations in a patient population with different severity levels. In a population of patients with prostate cancer, we can now distinguish (using different staging approaches, Gleason scores, etc.) between cancers of different risk levels [12]. Hence, how the lack of knowledge affects our severity assessment is not unique to at-risk populations not having developed a condition, but part and parcel of any severity assessment. However, there is a difference between assessing the severity of a developed condition, which is ranked on a continuous scale, and assessing risk, which is a dichotomous phenomenon. If an at-risk individual eventually develops the condition, severity is assessed in the same way as it is for patients who have had the condition from the beginning.

A counterargument could be that the lack of knowledge has too great an impact on severity assessments in some cases; e.g., when it concerns at-risk populations who have not yet developed a specific condition. Consider the following example:

#### Case 4

In population C, all 10 patients have developed condition Y, there is some variation in severity among these 10 patients (75, 78, 79, 82, 82, 82, 83, 85, 85, 97) and the mean severity is 82.8. In population D ,there is an uneven risk distribution of developing Y (ranging from 1 to 95%, but where we cannot identify which individuals have the respective risk levels) but with a mean risk of 10%. Regarding population D developing Y we expect a similar variation in severity as that for population C, but with a mean severity of 82.8. In the whole population D, we will have a mean severity of 8.28 at T_0_ (= mean risk X of mean severity for when Y is developed in population D).

In population C, we might argue that the patient with a severity of 97 will suffer somewhat from being an outlier in C, and should actually have a greater claim on resources than the rest of population C. However, she will be treated as if she had a severity of 82.8, since priority decisions are made at the group level, and we do not distinguish between different individuals at this level. Still, all patients in C are in the top rank of severity, implying great claims on resources—hence the disadvantage are likely to be rather small. In population D, on the other hand, the few patients with a 95% risk, will have a severity of 78.7, but they will be highly disadvantaged by the fact that they belong to a population where the majority have a very low risk of developing Y. In other words, these low-risk persons ‘dilute’ the severity for those persons at a high risk of developing condition Y. In line with the argument supporting the Norwegian approach, we might claim that taking risk into account will affect the priority of prevention too greatly.

Another counterargument against taking risk into account goes something like this. Let us assume that the level of severity decides what level of cost-effectiveness is acceptable to a healthcare system (this is how severity is used in Sweden, Norway, and the Netherlands) [1, 4, 5]. Following this, we would accept a lower level of cost-effectiveness—i.e., a higher willingness to pay (WTP) threshold, for more severe conditions than for less severe conditions. Consider Case 2 again:

#### Case 2

Population A (n = 10) at T_0_ is suffering from condition X at severity 100. Population B (n = 100) at T_0_ has a low risk of developing condition X at T_1_, with 10 individuals having developed condition X at T_1_.

In both populations we can potentially save 10 people from having a severity of 100. Why not accept the same WTP for both populations? This would imply that we accept different costs per treatment and per patient for the two populations, but the cost per effect (saved life, alleviated suffering, etc.) will be the same. This indicates that we value the suffering, risk of death, etc., for the individuals in A and some as-yet unidentified individuals in B equally, and those individuals in B developing X at T_1_ will of course be as bad off as the individuals in A are at T_0_ (if we disregard the time-sensitive effects of severity discussed later). One can envision that the 10 individuals in group B who will later develop the condition X are actually the same individuals as those in group A, only at an earlier time point (when they only had a low risk of developing the condition). Thus, when discussing the severity of a condition in the preventive situation, whether we should downgrade severity with reference to risk actually depends on which population we decide to be relevant. If we consider the entire population of B to be relevant, there are good arguments for downgrading severity in relation to risk. However, if we consider only the unidentified (at T_0_) individuals in B who will later (at T_1_) develop the condition, downgrading severity in relation to risk will not be applicable (as everyone in this subgroup will develop the condition). In contrast, the 90 individuals in population B who never develop the condition are irrelevant and will have a severity of 0 at any time point including T_0_ and T_1_. In essence, this argument could be related to a general requirement of formal equality in priority setting; i.e., treating equal cases alike,[13] and would imply that populations A and B (i.e., those still unidentified individuals in B who will develop condition X at T_0_) have the same severity. Let us change the case slightly:

#### Case 5

Population A (n = 10) at T_0_ is suffering from condition X at a severity of 100, implying both negative QoL impact and a 100% risk of dying in one month. Population E (n = 10) at T_0 is_ suffering from condition Z at a severity 75, implying the same negative QoL impact as population A, but with a 10% risk of dying in one month.

Following the proposed line of argument, we might claim that in both populations, people will experience the same level of negative impact on QoL and die within a month. So why should we not have the same WTP for these two groups with different conditions? If so, we must assume that they have the same severity, implying that we should not take the risk of future death into account when assessing severity. Now, if we do not take the risk of future death into account, how could death be taken into account at all? Two different alternatives come to mind—one is to consider time-to-death and the other considers life-expectancy in relation to cohort or population life expectancy. On the first alternative, the shorter the time to death, the higher the severity. On the second alternative, the more life lost in relation to cohort or population life-expectancy, the higher the severity. Looking again at population E in Case 5, individuals have a 10% risk of dying in one month, but they also have some risk of dying in a week, and a risk of dying in a year, or in several years—with different severities for each of the alternatives. If we cannot take risk into account, what reason do we have to choose one of the alternative deaths before any other death? Moreover, if we use cohort or population life-expectancy as a reference level, this is also dependent on risks—so how should this be defined? Therefore, not taking risks into account when assessing severity in a group of patients with an established condition will result in not being able to take death into account in the severity assessment. This is a conclusion that most would probably find somewhat absurd. Likewise, if we—via some test—can identify two separate populations within the same condition with the prognoses of A and E respectively, we would certainly conclude that the subgroups have different severities. If this analogy can be generalised to also include the prognosis of an at-risk population again depends on which population we consider to be relevant.

**In conclusion**, it seems there are intuitive reasons to allow severity to be risk-sensitive. We can support this intuition by referring to consistency, since if we want to take death into account in our severity assessment, we need to take the risk of dying into account. It seems difficult to assess severity without taking death into account. Still, obviously, we might have different intuitions concerning this and how we define which population is relevant will be important for the conclusion.

A counter argument against allowing severity to be risk-sensitive is also that a lack of knowledge will ‘dilute’ the severity in a population; however, this is true of any assessment of severity in a population. It might be a problem that in the preventive situation—i.e., in situations where risk distribution has a great variation in a population, combined with lack of knowledge, the dilution of severity might be too extensive. We will return to the question of whether dilution is too large in the last section on systemic effects. Let us now turn to the question of whether time should be taken into consideration.

### Arguments for and Against Time-Sensitive Severity

Even if we do not accept risk-sensitive severity or find it problematic, we could argue that we should take discount for time, meaning that when a condition will appear in the future instead of right now should affect our assessment of severity. It is documented that people tend to have a ‘now’ bias, prioritising a benefit that is operative now rather than a benefit that occurs later (and vice versa with costs)—what is often called a ‘pure time’ preference [14, 15]. More generally, in a health-economy, the discounting of future events is generally applied to costs and (to a large extent) also to benefits, based on different rationales besides pure time preference [14, 16].

Let us start with pure time-preferences and explore whether it is rational to allow them to affect how we assess severity of future conditions. As a starting point it is obvious that if you are suffering from a condition that is likely to kill you in a month, it is rational to prioritise that condition before a condition that will materialise in 20 years and kill you. More, generally, if you live in a highly insecure environment where the risk of dying imminently is high, it is rational to have a time-preference prioritising the present above the future. Hence, our time-preference has a reasonable evolutionary explanation.

Whether we should have a special interest in this future person we call *I* depends on the assumption that we are (in some way) identical to this future person—something we might question [17]. However, let us ignore this complication here, since when we are prioritising scarce healthcare resources we are mainly considering the distribution between different persons or groups. So, let us change the example:

#### Case 6

Person G is now suffering from a condition that has a 100% risk of killing her in one month. Person H (at an age as G minus 20 years) has a genetic condition that has a 100% risk of materialising i20 years from now, and then kill her in one month with 100% risk. Which of these conditions is worse?

Given this, is the severity of H’s condition less severe simply because it will happen 20 years from now? It is difficult to find any rational reason for why the death of a person now is worse than the death of another person in the same situation in 20 years. Rather, severity for these two persons can be assumed to be the same. There are ethical approaches advocating that we should care about persons who are at imminent risk of dying and be willing to spend great resources on these persons to save them. This is voiced in the so-called ‘rule of rescue’. However, the rule of rescue has been criticised for being an irrational or biased psychological response; and as a moral principle, is difficult to square with considerations of fairness or justice [18–21]. Furthermore, it has been argued that when concerning challenges affecting future generations—e.g., climate change—applying such a pure time preference would seem to have potentially devastating consequences and could be viewed as treating future people highly unfairly [14, 16, 22, 23].

Let us therefore focus on other reasons for discounting the future. In health economy, we find three further rationales or types of arguments for discounting the future that are relevant here, besides pure time preferences:

- insecurity about the future.
- growth in public health resources.
- potential systemic or consistency aspects for discounting (or not discounting) the future [16, 24].

The discussion on DALYs (Disability Adjusted Life Years) and discounting could give us a background for this analysis. DALYs are a measurement of the health losses of a population at any given time, given the current health-state in which they find themselves [20]. In the conceptual development of the DALY in the beginning of the 1990s, it was standardly assumed that future DALYs should be discounted (despite acknowledging a critical discussion concerning this [24]. However, this seems to have changed over time and in some recent uses of the construct, no discount rates have been applied [25]. Let us start by examining the first of the three above rationales—insecurity about the future.

### Uncertainty About Future Events Besides Developing the Condition

Since we have already dealt with uncertainty about whether a specific population will develop a specific condition, for the discussion we now assume that the following situation holds:

#### Case 7

Population I is, at T_0_, suffering from condition Z that has a 100% risk of killing them in one month. Population J will develop condition Z, with 100% risk of death at T_0_ + 20 years, and it will then (as far as we know) kill them in one month.

Assuming I and J have the same severity, if *nothing* happens in 20 years except for J developing condition Z, what could happen that would change this assessment of severity? We start by considering two aspects—that individuals in J might die from something else before condition Z materialises, and that new technology might be developed that will cure or otherwise reduce the impact of condition Z.

In population J, some will have died from other causes before condition Z materialises. However, the only important thing here is if there is a population that will have the condition Z in 20 years. Unless we, through technological development, have eradicated the consequences of the condition, or there is a radical shift in makeup of the population, we are likely to have population J at T_0_ + 20 years. Hence, let us focus on technological development. Generally, over time, technological development of treatments for specific conditions will reduce the severity of these conditions. In many areas, new health technologies have revolutionised the treatment of health problems, curing or eradicating previously highly-lethal conditions, turning lethal conditions into manageable QoL problems, reducing the QoL impact of conditions that previously caused massive suffering, etc. Hence, over time, technology is expected to reduce the severity of health problems [26]. There are factors that could counteract this development, such as antibiotic resistance^4^ or climate change, which might affect our ability to benefit from a number of existing technologies in surgery and the treatment of infectious diseases, etc. [27]. Still, generally, the future seems promising. Given time, most conditions will probably benefit from technological development. However, over a shorter time perspective, technology is developing at different paces within different fields. Currently, gene therapies, cell therapies, and immunotherapies are developing quickly in regard to rare conditions and cancers. In contrast, the development of effective treatment in relation to psychiatric diseases, ALS, etc. seems less promising at the moment [28, 29]. Hence, generally allowing technological development to affect the severity of future conditions might disadvantage conditions where there is little or no development within the foreseeable future. Hence, in a short time perspective, individualised discounting of severity depending on the research front-line seems to be the most rational choice. In a long-term perspective, a more general discounting of severity could be more appropriate.

#### Growth in the Public Health Level

**Fig. 2:**
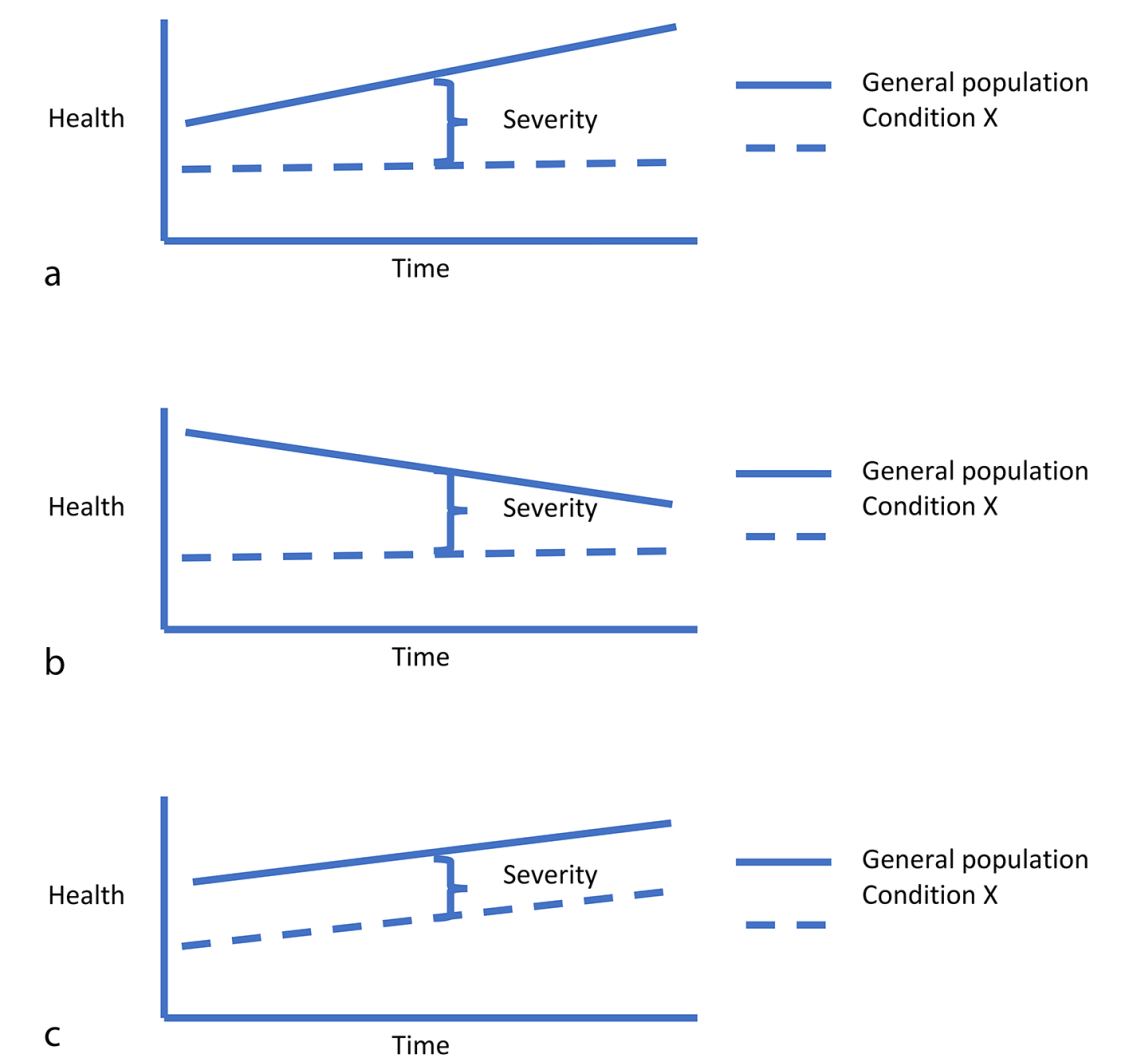
**a**. How severity may increase for a condition over time if the general health is growing and there is no technological development related to condition X. **b**. How severity may decrease for a condition over time if general health is diminishing due to catastrophic events (war, antibiotic resistance, climate change, etc.) and there is no technological development related to condition X. **c**. How severity may remain unchanged for a condition over time if general health is increasing in the same pace as the technological development related to condition X

When severity is assessed, the assessment is made in relation to some reference level of health in the population, often not the optimal health humans are capable of achieving but rather some accepted or average level of health in the specific population (or globally at this time) [24]. In the development of the DALY and choosing what this reference level should be (concerning life-expectancy), it was observed that population life expectancy is generally increasing over time (as is public health). An article by Wang et al. [30]. analysing global, regional, and local life-expectancy, relating 249 causes of death from 1980 to 2015, showed an overall increase in life-expectancy from 61.7 years to 71.8 years. This implies that looking at the lives lost using population-based life-expectancy at a specific time as a reference level, will differ from looking at a cohort-specific life-expectancy over time [24]. This general increase of the level of health in a society is dependent upon things like technological development but perhaps even more importantly, is also dependent on things like improvement of the general socioeconomic level and distribution in terms of education, employment, environmental factors, etc. Hence, a specific condition may remain largely unaffected by this general raise in population health. If so, the severity of this condition will increase over time (see Fig. 2a). As pointed out by Wang et al. (2009) there might be setbacks to this increasing trend; e.g. those related to HIV/AIDS, epidemics, war, etc. [30]. And we might add things like climate change or antibiotic resistance to such a list (Fig. 2b). Theoretically, this is an argument for not discounting future severity (rather the opposite) in situations where we expect population health to increase, but the technological development for a specific condition is not superior to the level of development of treatment for conditions in general.

Still, this might be of no real practical interest in relation to specific conditions. In the short timeframe where we can predict whether there will be technological development for a specific condition or not, we will probably not have reason to believe in a substantial improvement of public health. In a timeframe over which we can expect increasing public health, we will be unable to claim that there will be no technological development for the condition. However, we might, perhaps, over a shorter timeframe, predict a decrease in public health (due to climate change or antibiotic resistance or something analogous). If so, this is rather yet another argument for discounting future severity.

### Consistency Argument

One argument for having equal discounting rates for cost and effect is the consistency argument. Here in Attema et al. [16] (p. 747): “…two programs that are identical except for their timing. If one wants these identical programs to receive equal priority in decision making, this can only be accomplished by applying the same discount rate to costs and effects.”[31] Assume we find the consistency argument convincing and consider the following case:

#### Case 8

Population K is at T_0_ suffering from a condition Z that has a 100% risk of killing them in one month. There is cure for Z at a cost of 1000 € at T_0_. Population L will develop condition Z, with 100% risk, at T_0_ + 20 years, and it will then (as far as we currently know) kill them in one month. There is an intervention that will prevent L from developing Z at a cost of 1000€ at T_0_. We might assume population K = L in terms of the number of individuals.

Since these two programs are equal, according to the consistency argument, we should discount the cost and effect of the program for population L equally, in order not to disadvantage this program in relation to the program for population K. However, the consistency argument would then also tell us not to discount future severity, since that would disadvantage the program for L (with a lower severity, this program would have a lower priority). A problem with the consistency argument is that it presupposes that the monetary value of health is stable over time, which has been questioned [32].

In conclusion, the strongest argument for discounting severity over time and thus in prevention, is technological development. However, over a short timeframe, this will be different within different fields of healthcare, and a general discounting would disadvantage conditions where there is little hope of development in the near future. Counterarguments against discounting severity over time is that the reference level of public health generally increases over time. In specific fields where there is strong technological development, and we have a fairly short time perspective this is not likely to counter the impact of technology. On a larger time perspective, the relationship between public health and technology is much more difficult to assess, and even the level of public health might take a turn for the worse given, for example, antibiotic resistance and climate change [33]. Finally, the consistency argument also presents a potential counterargument against discounting future severity.

### Systemic Effects of Taking risk and Time into Account

The main argument for the Norwegians not allowing risk or time to affect the severity of future events was that it would systematically disadvantage prevention in a way that is not reasonable. This draws from the idea that preventing disease from occurring is generally better than treating developed disease, which to some extent is an open question. If we have access to very cost-effective treatments for a developed disease (basically curative treatment), this is likely to be better than less cost-effective prevention of such a disease. However, there might be cases when prevention is absolutely preferable. Consider a highly effective vaccination program for a serious and lethal contagious disease, for which there is only palliative treatment, once developed. The disease has a small risk of developing and spreading in an otherwise healthy population, but once developed will spread to a large part of the population. If we allow severity to be risk- and/or time-sensitive and do not allow severity to take into account the size of the affected population (which we normally do not), severity will be very low with a resulting low priority for the vaccination program. We find arguments to this extent within the discussion of discounting in health economy [34]. If so, this would be an unwanted systemic effect of risk- and/or time-sensitive severity.

### Summing Up

In analysing whether severity should be risk- and/or time-sensitive when applied in priority setting in healthcare, we have found that there is an argument for taking the risk of developing a condition into account, and the fact that we already do this when we take into account the risk of dying in the severity assessment of a developed condition will strengthen this argument.

If severity is discounted according to risk, this will ‘dilute’ severity; a dilution that is dependent on how well we are able to delineate the population under consideration, which in turn is dependent on the current level of knowledge. This is a general feature of all severity assessments of a population but will have a potentially farther reaching effect when we are considering primary prevention; i.e., large populations with a small mean risk to develop a specific condition. At a systemic level, this might make us de-prioritise effective preventive treatments in relation to acute, less effective treatments. A potentially promising way to de-emphasise such dilution is to apply a maximum risk approach, but such an approach needs to be developed more in detail before drawing any conclusions.

Another aspect of risk sensitivity is which population is relevant when assessing the severity. If we consider the entire population at risk at T_0_ to be the relevant population, we need to take the risk of developing the condition into consideration along with the issues regarding dilution of severity. If we, in terms of prevention, instead focus on the population of as-yet (at T_0_) unidentified individuals who will develop the condition at T_1_, risk will become irrelevant, and severity will not be risk sensitive. Whether severity is time sensitive will still be an issue, independent of which population is considered relevant in assessing severity.

We have found that the strongest argument for time-sensitive severity (or discounting future severity) is the future development of technology. However, on a short timescale, this will differ between different diagnoses, and we might have reason to individualise discounting based on this. On a large timescale, a more general discounting might be acceptable. On the other hand, this might be countered to some extent by technological development in general and increasing levels of public health, but the net-sum effect will be dependent on both the timescale and how public health develops. On a shorter timescale, technology is likely to trump public health development in conditions currently undergoing rapid progress in the development of effective therapies. A further counterargument against discounting severity in prevention is the consistency argument. Once again, there might be negative systemic effects to this that must be taken into consideration.

### Implications for Health Systems

Can we draw an overall conclusion from this? By only looking at severity in isolation, we seem to have some reason both for and against making it both risk- and time-sensitive (even if we have reason to individualise the latter depending on the type of condition if over a shorter timescale). However, quantifying risk-sensitivity in relation to level of risk will have far-reaching consequences for the priority of primary prevention in large populations with small general risks of developing conditions. That is, if severity is a central factor to consider in priority setting, when we apply risk and time-sensitivity to severity, primary prevention will be systematically down-prioritised in relation to interventions against manifest conditions. Since we have good reasons to try to prevent illness before it starts affecting our quality of life and life-expectancy (or ‘health’, in short), this would be an unfortunate implication. This will depend on how and to what extent primary prevention will be down-prioritised. It implies that we set a lower acceptable cost-effectiveness threshold for primary prevention, but still, most interventions will still make this threshold—this implication might be of more theoretical than practical importance. If, on the other hand, it will actually have a more far-reaching impact on access or preventive interventions, such systemic effects might be less acceptable. On the other hand, applying the same cost-effectiveness thresholds for primary prevention, since we do not apply risk- and time-sensitivity to severity, might imply the acceptance of the costs of some preventive interventions at a higher level at the expense of interventions for manifest conditions (or other preventive interventions). Especially for interventions where the cost is dependent on a price set on a market (as is the case for drugs) and this pricing is endogenous, this is an obvious risk. Therefore, there are systemic effects that we will have to consider, regardless of which strategy we decide to adopt.

If we find the arguments for risk-and time-sensitive severity convincing but want to mitigate the systemic effects, we could allow the discounting of future events to vary somewhat depending on the risk of suffering this future event. With a small risk of developing a future condition, we apply a somewhat higher discounting factor than we could in the case of a very high risk. To take the Swedish example, the general 3% discount factor could be applied to severity, and if the risk of developing the condition is very low, we might apply up to 5–6%. Alternatively, severity could be discounted only according to time-sensitivity if we instead accept that the risk level is irrelevant. Moreover, the systemic effects of this will be dependent on how severity is supposed to affect the rest of priority setting; e.g., the WTP level or the like. Looking at the discount rates of different countries, it seems there is no robust empirical support for the actual levels that are accepted; therefore such an approach might have a less robust basis [16].

## Data Availability

N/A.

## Footnotes

1 Z_current level_ is simply Z_potential level_ where risk is set to 1 and there is no discounting for time.

2 In both jurisdictions, the level of population risk and the relevant time will affect the assessment of effect and the incremental cost-effectiveness ratio of an intervention.

3 For brevity, we will talk in terms of *risk* when normally referring to *probability* in this text.

4 We owe this point to Ann Söderström.

## Code Availability

N/A.
